# UAV formation control design with obstacle avoidance in dynamic three-dimensional environment

**DOI:** 10.1186/s40064-016-2476-y

**Published:** 2016-07-19

**Authors:** Kai Chang, Yuanqing Xia, Kaoli Huang

**Affiliations:** School of Automation, Mechanical Engineering College, Shijiazhuang, 050003 China; School of Automation, Beijing Institute of Technology, Beijing, 100081 China

**Keywords:** Trajectory, Formation control, Assignment, Collision avoidance

## Abstract

This paper considers the artificial potential field method combined with rotational vectors for a general problem of multi-unmanned aerial vehicle (UAV) systems tracking a moving target in dynamic three-dimensional environment. An attractive potential field is generated between the leader and the target. It drives the leader to track the target based on the relative position of them. The other UAVs in the formation are controlled to follow the leader by the attractive control force. The repulsive force affects among the UAVs to avoid collisions and distribute the UAVs evenly on the spherical surface whose center is the leader-UAV. Specific orders or positions of the UAVs are not required. The trajectories of avoidance obstacle can be obtained through two kinds of potential field with rotation vectors. Every UAV can choose the optimal trajectory to avoid the obstacle and reconfigure the formation after passing the obstacle. Simulations study on UAV are presented to demonstrate the effectiveness of proposed method.

## Background

Rapid advances in computing, sensor, communication technologies have led to development of multiple aircrafts in dynamic three-dimensional environment. Formation control has been one of the most important research topics in multiple aircrafts systems due to its broad applications, including research and rescue missions, transportation, localization of chemical source, operational missions, to name of a few (Li et al. 2010; Waharte and Trigoni 2010; Maza et al. 2010).

For the multi UAV system, estimating and tracking the motion of a moving target is a crucial problem. The artificial potential field method is normally used to control a swarm of UAVs with obstacle avoidance (Khatib 1986). The attractive force leads the UAV to the desired position and the repelling force keeps UAV from the collisions with other UAVs or obstacles. The control force depends on the relation position of UAVs, targets and obstacles of the dynamic three-dimensional environment. The artificial potential field around the obstacle provides the repelling force in a typically small range. The attractive force effects in large range of the environment as long as it is observed.

A common problem with the artificial potential field method is the existence of local minima in the multi UAV system. It means that the repelling force is in the opposite direction of approaching UAV and the UAV will stick in local minima. A possible solution to this problem is to add a small disturbance in vertical direction when it is stuck (Chengqing et al. 2000). However, the UAV can not maintain the speed and this problem becomes complicated as the number of UAVs in the formation increases. In Masoud and Masoud (2002) the UAVs avoid the obstacles without containing local minimum points by using combination of the artificial potential fields and static fields. In this method, UAVs are navigated by generalized artificial potential field in an area which has known static obstacles.

In recent years, a number of various approaches for controlling a group of mobile robots to follow the track which aims to reach target point by avoiding obstacles in 2D (two-dimensional) space, see Rezaee and Abdollahi (2014), Hu and Feng (2010), Cui (2010). The collision and obstacle avoidance mechanism has been provided autonomously to ensure stability and robustness of the group by using this method. Furthermore, the tracks which aim to reach target points and avoid obstacles have been defined for the formation of UAVs in 3D (three-dimensional) space without effecting local minimums in Garcia-Delgado et al. (2012), Filippis et al. (2012). In Weihua and Go (2011), model predictive control (MMPC) method for UAVs formation coordination and obstacle avoidance for any shape and size of obstacles is introduced.

In this paper, the artificial potential field method is combined with rotational vectors and applied to formation control of UAVs. An UAV in formation is defined as the leader. The member UAVs follows the leader-UAV to track a moving target with obstacle avoidance in dynamic three-dimensional environment. By the attraction of the artificial potential field from the target, the leader will drive the formation to approach the target position. Each member-UAV will be connected with its neighbors and maintain in desired distance with neighbors. The moving trajectory of each member-UAV is controlled by the total potential field consisting of the attractive field of the leader and the repulsive fields of its neighbors. The potential fields with rotational vectors around the obstacles are divided into two kinds of potential fields, the potential fields parallel to *x*–*y* plane and the potential fields parallel to *y*–*z* plane. Each kind of potential field has two directions of rotational vectors. In the potential field, the rotational vectors adjust the direction of a UAV to lead it toward its target without being trapped in local minimum positions. This technique can choose the optimal path for each UAV and reconfigurate the formation for the swarm formation. The formation of UAVs track a moving target and keep the robustness and stability of the formation with obstacle and collision-avoidance by using this artificial potential field method.

The rest of paper is organized as follows: In second section, dynamics of unmanned vehicles model is defined. The proposed method of target tracking and formation configuration is presented in third section. Fourth section presented the obstacle avoidance method for unmanned vehicle formation. Fifth section gives the simulation results. Finally, some conclusions and ideas for future work are given in sixth section.

## Unmanned vehicle dynamical equations and control

**Fig. 1:**
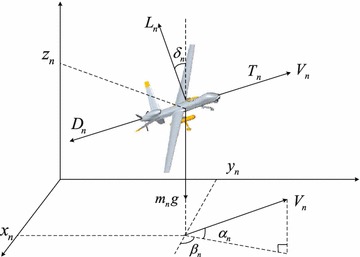
Unmanned aerial vehicle aerodynamic coordinate system model

First, we consider UAV’s dynamical equations which has been widely used in many literatures (Rezaee and Abdollahi 2013; Lin 2014; Wang and Xin 2013). The unmanned vehicle dynamical equations can be described by 3-DOF (degree of freedom) point mass model as follows (Wang and Xin 2013):

1 $$\begin{aligned} \dot{x}&= V\cos \alpha _n \cos \beta _n \nonumber \\ \dot{y}&= V\cos \alpha _n \sin \beta _n \nonumber \\ \dot{z}&= V\sin \alpha _n \nonumber \\ \dot{V}&= \frac{{T - D}}{m} - g\sin \alpha _n \nonumber \\ \dot{\alpha }_n&= \frac{{L\cos \delta _n - mg\cos \alpha _n }}{{mV}}\nonumber \\ \dot{\beta }_n&= \frac{{L\sin \delta _n }}{{mV\cos \alpha _n }} \end{aligned}$$

where *m* is UAV’s mass, *D* is the damping coefficient, *T* is the engine thrust, *g* is the gravitational acceleration, $({\dot{x}}, {\dot{y}}, {\dot{z}})$ is the velocity vector of UAV at coordinate axis direction in 3D space, *L* is the lift force, ${\alpha _n}$ is the flight path angle, ${\beta _n}$ is the heading angle, ${\delta _n}$ is banking angle, *V* is the air speed which is assume to be be equal to the ground speed in this paper. Figure 1 is shown the UAV aerodynamic coordinate system model.

The control inputs of UAV is engine thrust *T*, lift force *L*, and the banking angle ${\delta }$. The highly nonlinear UAV model (1) can be pre-linearized using feedback linearization as follows (Menon et al. 1999):

2 $$\begin{aligned} {\ddot{x}}&= {u_{x}}\nonumber \\ {\ddot{y}}&= {u_{y}}\nonumber \\ {\ddot{z}}&= {u_{z}} \end{aligned}$$

where $({u_{x}}, {u_{y}}, {u_{z}})$ is the virtual acceleration control inputs. The virtual control inputs are designed base on the linear model (2). The real control inputs can be obtained through the following equations

3 $$\begin{aligned} {\delta }&= {\tan ^{ - 1}}\left( {\frac{{{u_{y}}\cos {\beta } - {u_{x}}\sin {\beta }}}{{\left( {{u_{z}} + g} \right) \cos {\alpha } - \left( {{u_{x}}\cos {\beta } + {u_{y}}\sin {\beta }} \right) \sin {\alpha }}}} \right) \nonumber \\ {L}&= {m}\frac{{\left( {{u_{z}} + g} \right) \cos {\alpha } - \left( {{u_{x}}\cos {\beta } + {u_{y}}\sin {\beta }} \right) \sin {\alpha }}}{{\cos {\delta }}}\nonumber \\ {T}&= {m}\left( {\left( {{u_{z}} + g} \right) \sin {\alpha } + \left( {{u_{x}}\cos {\beta } + {u_{y}}\sin {\beta }} \right) \cos {\alpha }} \right) + {D} \end{aligned}$$

where $$\tan {\beta _n} = {\dot{y}}/{\dot{x}}$$ and $$\sin {\alpha _n} = {\dot{z}_n}/{V_n}$$.

## Formation control

In this section presents, an extended artificial potential field method is presented for a leader–follower formation of UAVs with obstacle avoidance in 3D environment.

### Control algorithm for each member-UAV

The motion of the member-UAV is driven by the total artificial force that consists of two components as follows:

4 $$\begin{aligned} u_n = \frac{{{f_{nc}} + {f_n}}}{m} \end{aligned}$$

where *m* is the mass of the *n*th UAV. The first component $f_{nc}$ is an attractive force to control the UAV to reach the spherical surface whose center is leader UAV. $f_{nc}$ can be described as:

5 $$\begin{aligned} {f_{nc}} = \left( {{f_{{x_{nc}}}},{f_{{y_{nc}}}},{f_{{z_{nc}}}}} \right) \end{aligned}$$

where

6 $$\begin{aligned} f_{x_{nc}}&= -{k_s}\left( {{x_n} - {x_l}} \right) \left( {{{\left( {{x_n} - {x_l}} \right) }^2}+ {{\left( {{y_n}-{y_l}} \right) }^2}+{{\left( {{z_n}-{z_l}} \right) }^2}-{{r_a}^2}} \right) \nonumber \\ f_{y_{nc}}&= - {k_s}\left( {{y_n} -{y_l}} \right) \left( {{{\left( {{x_n} - {x_l}} \right) }^2} + {{\left( {{y_n}-{y_l}} \right) }^2}+{{\left( {{z_n}-{z_l}} \right) }^2}-{{r_a}^2}} \right) \nonumber \\ f_{z_{nc}}&= - {k_s}\left( {{z_n} - {z_l}} \right) \left( {{{\left( {{x_n} - {x_l}} \right) }^2} + {{\left( {{y_n} - {y_l}} \right) }^2} + {{\left( {{z_n} - {z_l}} \right) }^2} - {{r_a}^2}} \right) \end{aligned}$$

where $(x_l,y_l,z_l)$ is the coordinate of the leader UAV. $k_s$ is the gain coefficient.

The second component $f_n$, which is the resultant force of repulsive forces of the UAVs, is created to arrange the unmanned vehicles evenly distributed on the spherical surface. We define leader-UAV as the center at $\left( {{x_l},{y_l},{z_l}} \right)$ and all UAVs have been negatively or positively charged. The repulsive forces affect on the unmanned vehicles which have identical electric charge. The control force that is defined in (5) keeping the UAVs on the spherical surface whose radius is $r_a$ and center is $\left( {{x_l},{y_l},{z_l}} \right)$. The unmanned vehicle reaches the equilibrium point when the resultant of repulsive forces tangent to the spherical surface acting on an UAV is zero. It means that the distances between UAVs are equal. Then, the goal is achieved. The repulsive force between two UAVs is defined as follows:

7 $$\begin{aligned} {f_{ni}} = {k_r}\frac{{{q_n}{q_i}}}{{r_{ni}^2}} \end{aligned}$$

where ${{q_n}}$ is electric quantity of the *n*th unmanned vehicle, ${{q_i}}$ is electric quantity of the *i*th unmanned vehicle, ${k_r}$ is the repulse constant coefficient, ${r_{ni}}$ is the distance between the *i*th and the *n*th unmanned vehicle. The amount of unmanned vehicle is *N*. Hence, the resultant of the repulsive forces from the other unmanned vehicles on the *n*th unmanned vehicle is given as follows:

8 $$\begin{aligned} {f_n} = {k_r}{q_n}\sum \limits _{i = 1,i \ne n}^N {\frac{{{q_i}}}{{r_{ni}^2}}} \end{aligned}$$

**Fig. 2:**
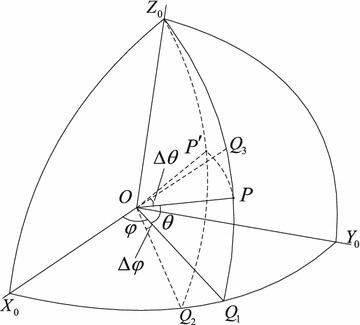
Direction of the unmanned vehicle movement

In Fig. 2, *P* is the initial position of UAV. $P'$ is the position of UAV after move. $Q_1$ is projection of *P* on the $X_0-O-Y_0$ plane. $Q_2$ is projection of $P'$ on the $X_0-O-Y_0$ plane. $Q_3$ is projection of $P^{\prime }$ on the $Z_0-O-Q_1$ plane. By decomposing the resultant force in three directions, component forces are in the *x*-axis direction, *y*-axis direction and the *z*-axis direction, respectively. It is shown in Fig. 2 as follows:

9 $$\begin{aligned} {f_{x_{n}}}&= {k_r}{q_n}\sum \limits _{i = 1,i \ne n}^N {\frac{{{q_n}{q_i}}}{{r_{ni}^2}}\cos {\theta _{ni}}\cos {\varphi _{ni}}} \nonumber \\ {f_{y_{n}}}&= {k_r}{q_n}\sum \limits _{i = 1,i \ne n}^N {\frac{{{q_n}{q_i}}}{{r_{ni}^2}}\cos {\theta _{ni}}\sin {\varphi _{ni}}} \nonumber \\ {f_{z_{n}}}&= {k_r}{q_n}\sum \limits _{i = 1,i \ne n}^N {\frac{{{q_n}{q_i}}}{{r_{ni}^2}}\sin {\theta _{ni}}} \end{aligned}$$

where

$$\begin{aligned} \sin {\theta _{ni}}&= \frac{{{z_n} - {z_i}}}{{\left| {{r_{ni}}} \right| }}\\ \cos {\theta _{ni}}&= \frac{{\sqrt{{{\left( {{x_n} - {x_i}} \right) }^2} + {{\left( {{y_n} - {y_i}} \right) }^2}} }}{{\left| {{r_{ni}}} \right| }}\\ \cos {\varphi _{ni}}&= \frac{{{x_n} - {x_i}}}{{\sqrt{{{\left( {{x_n} - {x_i}} \right) }^2} + {{\left( {{y_n} - {y_i}} \right) }^2}} }}\\ \sin {\varphi _{ni}}&= \frac{{{y_n} - {y_i}}}{{\sqrt{{{\left( {{x_n} - {x_i}} \right) }^2} + {{\left( {{y_n} - {y_i}} \right) }^2}} }}\\ {r_{ni}}&= \sqrt{{{\left( {{x_n} - {x_i}} \right) }^2} + {{\left( {{y_n} - {y_i}} \right) }^2} + {{\left( {{z_n} - {z_i}} \right) }^2}} \end{aligned}$$

Consider the *n*th UAV’s dynamical equation (2), the *n*th UAV’s dynamical equations based on the virtual structure can be rewritten as:

10 $$\begin{aligned} {u_{{x_n}}}&= \frac{{{f_{{x_{nc}}}} + {f_{{x_n}}}}}{m}\nonumber \\ {u_{{y_n}}}&= \frac{{{f_{{y_{nc}}}} + {f_{{y_n}}}}}{m}\nonumber \\ {u_{{z_n}}}&= \frac{{{f_{{z_{nc}}}} + {f_{{z_n}}}}}{m} \end{aligned}$$

Given the above discussion, the repulsive force that is proportional to $1/{r_{ni}}$ avoids the collision between unmanned vehicles. The control force $(u_{x_n},u_{x_n},u_{x_n})$ in (10) leads the UAVs toward the equilibrium points on the spherical surface.

### Control algorithm for the UAV-leader

The motion of the UAV-leader is driven by the attractive force to reach a moving target. The position of leader UAV $p_l=(x_l,y_l,z_l)$. Consider the target position is $p_t=(x_t,y_t,z_t)$, the attractive force can be stated as follow:

if $$r < d$$

11 $$\begin{aligned} {f_{{x_{att}}}}&= - {k_t}\left( {{x_l} - {x_t}} \right) \nonumber \\ {f_{{y_{att}}}}&= - {k_t}\left( {{y_l} - {y_t}} \right) \nonumber \\ {f_{{z_{att}}}}&= - {k_t}\left( {{z_l} - {z_t}} \right) \end{aligned}$$

else

$$\begin{aligned} {f_{x_{att}}}&= - {k_t}\left( {{x_l} - {x_t}} \right) \frac{{{d_{lt}}}}{{{r_t}}}\nonumber \\ {f_{y_{att}}}&= - {k_t}\left( {{y_l} - {y_t}} \right) \frac{{{d_{lt}}}}{{{r_t}}}\nonumber \\ {f_{z_{att}}}&= - {k_t}\left( {{z_l} - {z_t}} \right) \frac{{{d_{lt}}}}{{{r_t}}} \end{aligned}$$

where $k_t$ is the positive constants. $d_{lt}$ is the distance between target and the leader UAV. ${d_{lt}} = \sqrt{{{\left( {{x_t} - {x_l}} \right) }^2} + {{\left( {{y_t} - {y_l}} \right) }^2} + {{\left( {{z_t} - {z_l}} \right) }^2}}. r_t$ is defined as the range of the target.

The relative velocity among the leader and the target is added as a damping force to control the leader-UAV when it approaches the range of the target position. The damping force force is proposed as follows:

12 $$\begin{aligned} {f_{x_{dam}}}&= - {k_m}\left( {{{\dot{x}}_l} - {{\dot{x}}_t}} \right) \nonumber \\ {f_{y_{dam}}}&= - {k_m}\left( {{{\dot{y}}_l} - {{\dot{y}}_t}} \right) \nonumber \\ {f_{z_{dam}}}&= - {k_m}\left( {{{\dot{z}}_l} - {{\dot{z}}_t}} \right) \end{aligned}$$

where $k_m$ is the positive amplification coefficient. The control force is the resultant force of the attractive force and damp force

13 $$\begin{aligned} {f_{x_{l}}}&= {f_{x_{att}}} + {f_{x_{dam}}}\nonumber \\ {f_{y_{l}}}&= {f_{y_{att}}} + {f_{y_{dam}}}\nonumber \\ {f_{z_{l}}}&= {f_{z_{att}}} + {f_{z_{dam}}} \end{aligned}$$

### Stability analysis

In order to analyze the stability of the formation, we analyze the a UAV at the equilibrium point. It means that ${f_n}$ is zero. Lemma 3.1 provides a trajectory that UAV moves to a spherical surface whose center is $({x_c}, {y_c}, {z_c})$ and radius is $r_a$. It is stable when the UAV reaches the spherical surface. In other words velocity of UAV will be zero.

#### **Lemma 1**

*The desire trajectory of UAV satisfies*

14 $$\begin{aligned} \dot{x}&= -\left( {x - {x_c}} \right) \left( {{{\left( {x - {x_c}} \right) }^2} + {{\left( {y- {y_c}} \right) }^2} + {{\left( {z - {z_c}} \right) }^2} - {{r_a}^2}} \right) \nonumber \\ \dot{y}&= -\left( {y - {y_c}} \right) \left( {{{\left( {x - {x_c}} \right) }^2} + {{\left( {y - {y_c}} \right) }^2} + {{\left( {z - {z_c}} \right) }^2} - {{r_a}^2}} \right) \nonumber \\ \dot{z}&= -\left( {z - {z_c}} \right) \left( {{{\left( {x - {x_c}} \right) }^2} + {{\left( {y - {y_c}} \right) }^2} + {{\left( {z - {z_c}} \right) }^2} - {{r_a}^2}} \right) \end{aligned}$$

*where*$$(x, y, z) \ne (x_c, y_c, z_c)$$.

#### *Proof*

By inserting ${r^2} = {\left( {x - {x_c}} \right) ^2} + {\left( {y - {y_c}} \right) ^2} + {\left( {z - {z_c}} \right) ^2}, \varphi = \arctan \mathrm{}\left( {{{\left( {y - {y_c}} \right) } \big / {\left( {x - {x_c}} \right) }}} \right)$ and $\theta = \arctan \left( {{{\left( {z - {z_c}} \right) } \Bigg / {\left( {\sqrt{{{\left( {y - {y_c}} \right) }^2} + {{\left( {x - {x_c}} \right) }^2}} } \right) }}}\right)$ into Eq. (6). $\varphi$ and $\theta$ are shown in Fig. 2. The equations can be achieved as follows:

15 $$\begin{aligned} \dot{r} = - r\left( {{r^2} - {{r_a}^2}} \right) \;\;\;\;\dot{\theta }= 0\;\;\dot{\varphi }= 0 \end{aligned}$$

To prove the stability of UAV arriving at the spherical surface, it is obvious that $\dot{\theta }$ and $\dot{\varphi }$ converge to zero when $r={r_a}$, we define the error $\varepsilon =r-{r_a}$ and choose the following Lyapunov function candidate:

16 $$\begin{aligned} V\left( \varepsilon \right) = {\varepsilon ^2} \end{aligned}$$

The derivative of the Lyapunov function (4) is given by

17 $$\begin{aligned} \dot{V}\left( \varepsilon \right) = 2\varepsilon \dot{r} \end{aligned}$$

Substituting (3) into (5), we have

18 $$\begin{aligned} \dot{V}\left( \varepsilon \right) = - 2{\varepsilon ^2}r\left( {r +{r_a} } \right) \end{aligned}$$

Because $(x, y, z) \ne (x_c, y_c, z_c)$, it can be deuced that $r>0$. It is obvious that $\dot{V}\left( \varepsilon \right) \le 0$. Since $V\left( \varepsilon \right) = \mathrm{{0}}$ only if $r={r_a}$, it follows that velocity is non-increasing, $\dot{\theta }=0$, $\dot{\varphi }=0$ and $V\left( \varepsilon \right)$ is bounded. In other words, UAV’s trajectory converges to the spherical surface. Unmanned vehicle will have no rotational motion when arrives at the spherical surface.$\square$

## Obstacle avoidance

In this section, a novel method of obstacle avoidance for single UAV is presented. The strategy for formation obstacle avoidance is also proposed.

### Obstacle avoidance

We consider the trajectory tracking problem of UAV in dynamic 3-D environment. The position of UAV is available. We assume that obstacles can be detected by the UAV vision sensor. Without loss of generality, the obstacle can be considered as a rectangular solid $\left( {{x_o} \pm {v_1},{y_o} \pm {v_2},{z_0} \pm {v_3}} \right)$. Where $\left( {{x_o},{y_o},{z_0}} \right)$ is the center of obstacle. $\left( { \pm {v_1}, \pm {v_2}, \pm {v_3}} \right)$ are its vertices which parallel to x-, y-, z-axis respectively. The potential field covers the obstacles with the minimum volume that satisfies

19 $$\begin{aligned} \frac{1}{{3v_1^2}}{\left( {x - {x_o}} \right) ^2} + \frac{1}{{3v_2^2}}{\left( {y - {y_o}} \right) ^2} + \frac{1}{{3v_3^2}}\left( {z - {z_o}} \right) = 1 \end{aligned}$$

**Fig. 3:**
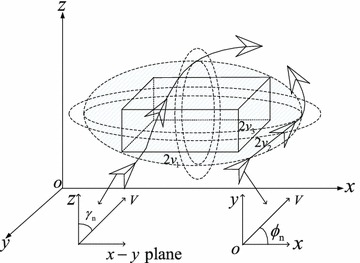
The potential fields with rotational vectors around an obstacle

The potential field method depends on repulsive force. When the repulsive force is in the opposite direction of approaching UAV, the UAV will stick in a local minimum position. To avoid this condition, rotational vectors are added to adjust the direction of the UAV. The potential fields with rotational vectors which cover this ellipsoid can be divided into two kinds of potential fields: the potential field which is parallel to x–y plane with rotational vectors and the potential field which is parallel to y–z plane with rotational vectors. It is depicted in Fig. 3. The desired trajectories which are effected by two kinds of potential fields with rotational vectors respectively are shown in Fig. 3.

Where ${\gamma _n}$ and ${\phi _n}$ are the velocity angles that take affect respectively in two kinds of potential fields.

20 $$\begin{aligned} {\phi _n}&= \arctan \left( {\dot{y},\dot{x}} \right) \nonumber \\ {\gamma _n}&= \arctan \left( {{\dot{z}},{{\sqrt{{{\dot{x}}^2} + {{\dot{y}}^2}} }}} \right) \end{aligned}$$

The trajectories of the UAV which only be effected by the rotational vectors in the potential fields parallel to *x*–*y* plane can be divided into two directions: clockwise direction and counterclockwise direction.

21 $$\begin{aligned} \dot{x}&= \frac{{v_2} }{{{v_1} }}\left( {x - {x_o}} \right) \nonumber \\ \dot{y}&= - \frac{ {v_1} }{{v_2}}\left( {y - {y_o}} \right) \mathrm{{\ \ in\ clockwise\ direction}}\nonumber \\ \dot{z}&= 0\end{aligned}$$

22 $$\begin{aligned} \dot{x}&= - \frac{{v_2}}{{v_1}}\left( {x - {x_o}} \right) \nonumber \\ \dot{y}&= \frac{{v_1}}{{v_2}}\left( {y - {y_o}} \right) \mathrm{{\ \ in\ counterclockwise\ direction}}\nonumber \\ \dot{z}&= 0 \end{aligned}$$

The trajectories of UAV which only be effected by the rotational vectors in the potential fields parallel to *y*–*z* plane can be divided into two directions: upward direction and downward direction.

23 $$\begin{aligned} \dot{x}&= \frac{{{v_1}{v_2}}}{{{v_3}\sqrt{{v_1}^2 + {v_2}^2} }}\left( {z - {z_o}} \right) \cos \left( \phi _n \right) \nonumber \\ \dot{y}&= \frac{{{v_1}{v_2}}}{{{v_3}\sqrt{{v_1}^2 + {v_2}^2} }}\left( {z - {z_o}} \right) \sin \left( \phi _n \right) \mathrm{{\ \ in\ upward\ direction}}\nonumber \\ \dot{z}&= - \frac{{{v_3}\sqrt{{v_1}^2 + {v_2}^2} }}{{{v_1}{v_2}}}\left( {x - {x_o}} \right) \cos \left( \phi _n \right) + \frac{{{v_3}\sqrt{{v_1}^2 + {v_2}^2} }}{{{v_1}{v_2}}}\left( {y - {y_o}} \right) \sin \left( \phi _n \right) \end{aligned}$$

24 $$\begin{aligned} \dot{x}&= -\frac{{{v_1}{v_2}}}{{{v_3}\sqrt{{v_1}^2 + {v_2}^2} }}\left( {z - {z_o}} \right) \cos \left( \phi _n \right) \nonumber \\ \dot{y}&= - \frac{{{v_1}{v_2}}}{{{v_3}\sqrt{{v_1}^2 + {v_2}^2} }}\left( {z - {z_o}} \right) \sin \left( \phi _n \right) \;\;\mathrm{{in}}\;\mathrm{{downward}}\;\mathrm{{direction}}\nonumber \\ \dot{z}&= \frac{{{v_3}\sqrt{{v_1}^2 + {v_2}^2} }}{{{v_1}{v_2}}}\left( {x - {x_o}} \right) \cos \left( \phi _n \right) + \frac{{{v_3}\sqrt{{v_1}^2 + {v_2}^2} }}{{{v_1}{v_2}}}\left( {y - {y_o}} \right) \sin \left( \phi _n \right) \end{aligned}$$

### Obstacle avoidance path optimization

In this section, the strategy of avoidance obstacles for formation is studied and the control force for single UAV is presented. We desire the rotational vectors effect when an UAV enter the range of the obstacle and be enlarged when the UAV is closer to the obstacle. The range of obstacle is denoted by $r_v$. The distance between the UAV to the obstacle is satisfying

25 $$\begin{aligned} {r_o} = \sqrt{{{\left( {x - {x_o}} \right) }^2} + {{\left( {y - {y_o}} \right) }^2} + {{\left( {z - {z_o}} \right) }^2}} \end{aligned}$$

The control force for avoidance obstacle can be stated as follow:

26 $$\begin{aligned} {f_{nr}} = ({f_{{x_{nr}}}},{f_{{y_{nr}}}},{f_{{z_{nr}}}}) \end{aligned}$$

Therefore the control force can be stated as follows:

if $${r_a} < {r_o}$$

$$\begin{aligned} {f_r} = {f_{desire}} + \frac{{\left| {{f_{desire}}} \right| {f_{nr}}}}{{r_o^2}}\left( {\frac{1}{{{r_o}}} - \frac{1}{{{r_v}}}} \right) \end{aligned}$$

else

27 $$\begin{aligned} {f_r} = {f_{desire}} \end{aligned}$$

where ${f_{desire}} = \left( {{f_{x_l}},{f_{y_l}},{f_{z_l}}} \right)$ for leader-UAV and ${f_{desire}} = \left( {{f_{{x_{nc}}}} + {f_{{x_n}}},{f_{{y_{nc}}}} + {f_{{y_n}}},{f_{{z_{nc}}}} + {f_{{z_{_n}}}}} \right)$ for member UAVs.

**Fig. 4:**
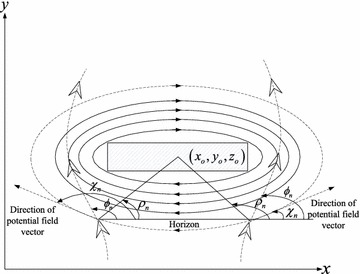
The trajectories in clockwise and counterclockwise direction respectively on x–y plane base on potential field with rotational vectors

Based on the trajectories in clockwise and counterclockwise direction which are effected by potential field with rotational vectors on x–y plane, the control force for avoidance obstacle can be designed as follows:

28 $$\begin{aligned} {f_{{x_{rxy}}}}&= {k_o}\frac{{{v_2}}}{{{v_1}}}\left( {y - {y_o}} \right) \nonumber \\ {f_{{y_{rxy}}}}&= - {k_o}\frac{{{v_1}}}{{{v_2}}}\left( {x - {x_o}} \right) {\hbox { in clockwise direction}}\nonumber \\ {f_{{z_{rxy}}}}&= 0 \end{aligned}$$

or

29 $$\begin{aligned} {f_{{x_{rxy}}}}&= - {k_o}\frac{{{v_2}}}{{{v_1}}}\left( {y - {y_o}} \right) \nonumber \\ {f_{{y_{rxy}}}}&= {k_o}\frac{{{v_1}}}{{{v_2}}}\left( {x - {x_o}} \right) {\hbox { in counterclockwise direction}}\nonumber \\ {f_{{z_{rxy}}}}&= 0 \end{aligned}$$

where $k_o$ is the gain coefficient. The rotational vectors in clockwise and counterclockwise direction are depicted in Fig. 4.

${\rho _n}$ is the angle between line linking the UAV and the center of gravity of the obstacle and the horizontal axis. ${\chi _n}$ is the direction of potential field vector. ${\phi _n}$ is the move direction of UAV. It can be obtained as follows:

30 $$\begin{aligned} {\phi _n}&= \arctan \left( {\dot{y},\dot{x}} \right) \nonumber \\ {\chi _n}&= \arctan \left( { - v_1^2{x_o},v_2^2{y_o}} \right) \nonumber \\ {\rho _n}&= \arctan \left( {{y_o} - y,{x_o} - x} \right) \end{aligned}$$

The direction of rotational vectors around the obstacle satisfies

if ${\phi _n} \ge {\rho _n}$ rotational vectors will be in clockwise direction.

if ${\phi _n} < {\rho _n}$ rotational vectors will be in counterclockwise direction.

Based on the trajectories in upward and downward direction which are effected by potential field with rotational vectors on y–z plane, the control force for avoidance obstacle can be designed as follows:

31 $$\begin{aligned} {f_{rxyz}}&= {k_o}\frac{{{v_1}{v_2}}}{{{v_3}\sqrt{{v_1}^2 + {v_2}^2} }}\left( {z - {z_o}} \right) \cos \left( {{\phi _n}} \right) \nonumber \\ {f_{ryyz}}&= {k_o}\frac{{{v_1}{v_2}}}{{{v_3}\sqrt{{v_1}^2 + {v_2}^2} }}\left( {z - {z_o}} \right) \sin \left( {{\phi _n}} \right) \;\;\mathrm{{in}}\;\mathrm{{upward}}\;\mathrm{{direction}}\nonumber \\ {f_{rzyz}}&= - {k_o}\frac{{{v_3}\sqrt{{v_1}^2 + {v_2}^2} }}{{{v_1}{v_2}}}\left( {x - {x_o}} \right) \cos \left( {{\phi _n}} \right) + \frac{{{v_3}\sqrt{{v_1}^2 + {v_2}^2} }}{{{v_1}{v_2}}}\left( {y - {y_o}} \right) \sin \left( {{\phi _n}} \right) \end{aligned}$$

or

32 $$\begin{aligned} {f_{rxyz}}&= -{k_o}\frac{{{v_1}{v_2}}}{{{v_3}\sqrt{{v_1}^2 + {v_2}^2} }}\left( {z - {z_o}} \right) \cos \left( {{\phi _n}} \right) \nonumber \\ {f_{ryyz}}&= -{k_o}\frac{{{v_1}{v_2}}}{{{v_3}\sqrt{{v_1}^2 + {v_2}^2} }}\left( {z - {z_o}} \right) \sin \left( {{\phi _n}} \right) \;\;\mathrm{{in}}\;\mathrm{{downward}}\;\mathrm{{direction}}\nonumber \\ {f_{rzyz}}&= {k_o}\frac{{{v_3}\sqrt{{v_1}^2 + {v_2}^2} }}{{{v_1}{v_2}}}\left( {x - {x_o}} \right) \cos \left( {{\phi _n}} \right) + \frac{{{v_3}\sqrt{{v_1}^2 + {v_2}^2} }}{{{v_1}{v_2}}}\left( {y - {y_o}} \right) \sin \left( {{\phi _n}} \right) \end{aligned}$$

**Fig. 5:**
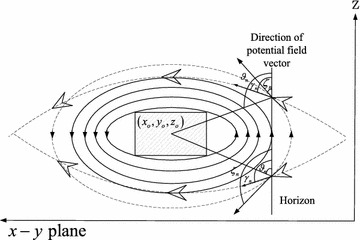
Rotational field with rotational vectors in upward direction and the downward in clockwise and counterclockwise direction respectively on y–z plane

The rotational vectors in upward and downward direction can be depicted in Fig. 5.

${\vartheta _n}$ is the angle between line linking the UAV and the center of gravity of the obstacle and the horizontal axis. ${\zeta _n}$ is the direction of potential field vector. ${\gamma _n}$ is the move direction of UAV. It can be obtained as follows:

33 $$\begin{aligned} {\gamma _n}&= \arctan \left( {\dot{z},\sqrt{{{\dot{x}}^2} + {{\dot{y}}^2}} } \right) \nonumber \\ {\zeta _n}&= \arctan \left[ \frac{{{v_1}{v_2}}}{{{v_3}\sqrt{v_1^2 + v_2^2} }}\left( {z - {z_o}} \right) ,\right. \nonumber \\&- \frac{{{v_3}\sqrt{v_1^2 + v_2^2} }}{{{v_1}{v_2}}}\left( {x - {x_o}} \right) \cos \left( \phi \right) - \frac{{{v_3}\sqrt{v_1^2 + v_2^2} }}{{{v_1}{v_2}}}\left( {y - {y_o}} \right) \sin \left( \phi \right) \nonumber \\ {\vartheta _n}&= \arctan \left( {{z_o} - z,\sqrt{{{\left( {{x_o} - x} \right) }^2} - {{\left( {{y_o} - y} \right) }^2}} } \right) \end{aligned}$$

The direction of rotational vectors around the obstacle satisfies

if ${\gamma _n} \ge {\vartheta _n}$ rotational vectors will be in downward direction.

if ${\gamma _n} < {\vartheta _n}$ rotational vectors will be in upward direction.

Now,we consider the strategy of avoidance obstacle for formation. During the formation maneuver, The control force for avoidance obstacle can be obtained by compare $\left| {{\gamma _n} - {\zeta _n}} \right|$ with $\left| {{\phi _n} - {\chi _n}} \right|$

if $$\mathrm{{ }}\left| {{\phi _n} - {\chi _n}} \right| < \left| {{\gamma _n} - {\zeta _n}} \right| \mathrm{{ }}$$

34 $$\begin{aligned} {f_{{x_{nr}}}}&= {f_{{x_{rxy}}}}\nonumber \\ {f_{{y_{nr}}}}&= {f_{{y_{rxy}}}}\nonumber \\ {f_{{z_{nr}}}}&= {f_{{z_{rxy}}}} \end{aligned}$$

else

35 $$\begin{aligned} {f_{{x_{nr}}}}&= {f_{{x_{ryz}}}}\nonumber \\ {f_{{y_{nr}}}}&= {f_{{y_{ryz}}}}\nonumber \\ {f_{{z_{nr}}}}&= {f_{{z_{ryz}}}} \end{aligned}$$

The control force for avoidance obstacle can be normalized and modified

36 $$\begin{aligned} {f_{nr}} = \left( {\frac{{{f_{{x_{nr}}}}}}{{\left\| {{f_{nr}}} \right\| }},\frac{{{f_{{y_{nr}}}}}}{{\left\| {{f_{nr}}} \right\| }},\frac{{{f_{{z_{nr}}}}}}{{\left\| {{f_{nr}}} \right\| }}} \right) \end{aligned}$$

## Simulation result

This section shows the performance of the proposed approaches in different scenarios. In Scenario 1, the leader UAV tracks the target position by using provided control algorithm.

### Scenario 1

**Fig. 6:**
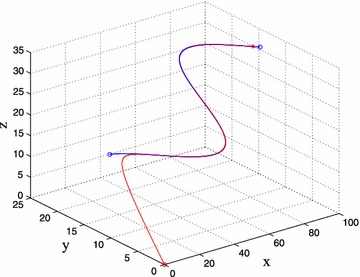
Unmanned vehicle flying in Scenario 1

Consider the target as a moving point in the free space. The initial position is (30, 20, 10), and the initial position of the leader is (0, 0, 0). The trajectory of target depends on (13) can be described as $f_{xt} = 10,f_{yt} = 10\times \sin \left( {1/8{x_v}} \right) ,f_{zv} = 2$. The simulation result in Fig. 6 shows that while reaching to the trajectory of the target the leader UAV always kept stable.

### Scenario 2

**Fig. 7:**
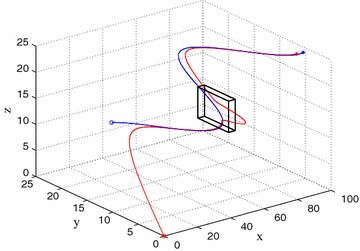
Unmanned vehicle formation flying in Scenario 2

Consider an obstacle with vertices located in ($20\pm 2,20\pm 3,10\pm 3$). The leader UAV switches to obstacle avoidance mode when it is close to the obstacle. The leader UAV selects the optimal trajectory, therefore it can catch up the target rapidly with a smoothly trajectory. Figure 7 shows the scenario of leader UAV flying with obstacle avoidance.

### Scenario 3

**Fig. 8:**
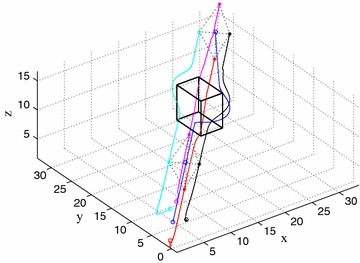
Unmanned vehicle formation flying in Scenario 3

Consider four member UAVs and a leader UAV, the initial position of member UAVs are as follows: ${p_1} = \left( {0.5,1,2.5} \right) ,{p_2} = \left( {3.5,3.5,7} \right) ,{p_3} = \left( {2, - 1,6.5} \right) ,{p_4} = \left( {2,3,6.5} \right) \mathrm{{ }}.$ The initial position of the leader UAV is ${p_L} = \left( {1,1,5.5}\right)$ and the obstacle with vertices is located in ${p_o} = \left( {20 \pm 2,20 \pm 3,10 \pm 3} \right)$. Without loss the generality, we set $M = 1,D = 1, k_r = 5, k_s = 5, k_t = 2, k_m = 1.5, {r_a} = 2.$ Simulation results depicted in Fig. 8 show that the organization of five UAVs is influenced by obstacle in 3D environment. The swarm avoids obstacle effectively and reconfigure the formation after avoiding the obstacle. The algorithm of the formation control, the leader-following motion of the formation, the obstacles avoidance, the collision avoidance between UAVs in the formation and the stability of the formation while moving are verified by simulation.

**Fig. 9:**
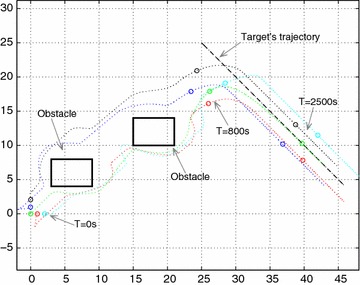
Unmanned vehicle formation flying in Scenario 4

### Scenario 4

The simulation case for a swarm of UAVs tracks the target with two obstacles, the initial position of the member UAVs are as follows: ${p_1} = \left( {1,0,6} \right) ,{p_2} = \left( {0,1,6} \right) ,{p_3} = \left( {0,2,6} \right) ,{p_4} = \left( {2,0,6} \right)$. The initial position of the leader UAV is ${p_L} = \left( {0,0,6} \right)$. The obstacles with vertices are located in ${p_{o1}} = \left( {6 \pm 3,6 \pm 2, 6 \pm 2} \right) , {p_{o2}} = \left( {18 \pm 3,12 \pm 2,6 \pm 2} \right)$. Figure 9 shows that every UAV can choose the optimal trajectory without requiring specific order or desire position in the formation. Obstacle avoidance is achieved successfully at t = 800 s. The formation of UAVs is rebuilt and maintained while the formation tracks a moving target.

## Conclusion

In this paper, an formation control approach has been addressed for multi-UAV systems to track a moving target under the drive of a UAV-leader based on the artificial potential field method combined with rotational vectors. The UAVs were able to configure the formation easily and quickly based on the attractive artificial potential field and move to the targets position. The repulsive forces kept UAVs from collision with each other during the maneuver. The effectiveness of the algorithm was verified through the simulations. Furthermore, exploring more features of the proposed method, we will focus on controlling the formation of UAVs in specific shape as the future direction of this research.
